# Linking Splicing to Pol II Transcription Stabilizes Pre-mRNAs and Influences Splicing Patterns

**DOI:** 10.1371/journal.pbio.0040147

**Published:** 2006-05-02

**Authors:** Martin J Hicks, Chin-Rang Yang, Matthew V Kotlajich, Klemens J Hertel

**Affiliations:** **1**Department of Microbiology and Molecular Genetics, University of California Irvine, Irvine, California, United States of America; **2**Institute for Genomics and Bioinformatics, University of California Irvine, Irvine, California, United States of America; University of WisconsinUnited States of America

## Abstract

RNA processing is carried out in close proximity to the site of transcription, suggesting a regulatory link between transcription and pre-mRNA splicing. Using an in vitro transcription/splicing assay, we demonstrate that an association of RNA polymerase II (Pol II) transcription and pre-mRNA splicing is required for efficient gene expression. Pol II-synthesized RNAs containing functional splice sites are protected from nuclear degradation, presumably because the local concentration of the splicing machinery is sufficiently high to ensure its association over interactions with nucleases. Furthermore, the process of transcription influences alternative splicing of newly synthesized pre-mRNAs. Because other RNA polymerases do not provide similar protection from nucleases, and their RNA products display altered splicing patterns, the link between transcription and RNA processing is RNA Pol II-specific. We propose that the connection between transcription by Pol II and pre-mRNA splicing guarantees an extended half-life and proper processing of nascent pre-mRNAs.

## Abstract 2

A novel in vitro method to study transcription and splicing leads to the proposal that linking transcription by Pol II and pre-mRNA splicing guarantees an extended half-life and proper processing of nascent pre-mRNAs.

## Introduction

Pre-mRNA splicing is a fundamental process required for the expression of most metazoan genes. It is carried out by the spliceosome that catalyzes the removal of non-coding intronic sequences to assemble exons into mature mRNA prior to export and translation [ 1]. Through the differential joining of coding sequences, alternative splicing generates multiple isoforms from a single gene, exponentially enriching the proteomic diversity of higher eukaryotic organisms [ 2– 4]. Crucial to understanding the pathway leading to this diversification are insights into the fundamental mechanisms that ensure efficient pre-mRNA splicing.

The generation of pre-mRNAs by RNA polymerase II (Pol II) has recently been demonstrated to influence pre-mRNA splicing patterns [ 5– 8]. These and many other biochemical and functional studies support the notion that the steps of pre-mRNA processing (capping, splicing, and polyadenyation) are functionally coupled to RNA Pol II transcription [ 7, 9– 12]. Thus, transcription and pre-mRNA processing are likely to be carried out in close proximity, possibly giving rise to higher order complexes. An essential component for connecting transcription and pre-mRNA processing is the C-terminal domain (CTD) of RNA Pol II [ 13]. For example, the efficient addition of a 7 methylguanosine triphosphate 5′ cap has been shown to depend on the presence of a fully hyperphosphorylated form of Pol II [ 14]. In fact, phosphorylated transcription complexes and recombinant CTD associate directly with guanyl-transferases [ 15– 17]. Similarly, cleavage at the poly(A) site depends on the CTD of Pol II [ 13, 18].

While the evidence for a physical link between transcription and splicing is not as substantial as for capping and polyadenylation factors [ 7, 11], several reports have provided compelling kinetic evidence to support functional coupling between transcription and splicing. For example, the identity of the transcription promoter influences alternative splice-site choice [ 5, 6, 19– 21]. In addition, pre-mRNA splicing efficiency can be increased significantly in vitro by the addition of phosphorylated Pol II [ 22]. These results support a model in which splicing factors associate with the Pol II transcription complex close to the promoter. As a consequence, differences in promoter structure could lead to differences in the splicing factors recruited to the transcription machinery. In an alternative model, the kinetics of Pol II transcription influence alternative splicing [ 23]. As Pol II polymerizes in a strict 5′–3′ direction, alternative exons are made prior to or after the synthesis of competing downstream or upstream exons. Accordingly, the relative timing of producing competing exons could bring about changes in the splice pattern. Strong support for this proposal stems from experiments testing the effects of elongation pause sites, different classes of transcription activators, and Pol II elongation mutants [ 8, 24– 27].

Independent of the mechanism, the connection between transcription by Pol II and pre-mRNA processing appears to provide a significant advantage to achieve high levels of gene expression. To gain insights into the biochemical and kinetic nature that govern the temporal link between transcription and pre-mRNA splicing, we have optimized an in vitro system that simultaneously carries out transcription and splicing. We demonstrate that integrating the two events, pre-mRNA splicing and transcription by Pol II, ensures efficient processing. This is achieved because the increased local concentration of the splicing machinery protects the newly synthesized pre-mRNA from degradation. Therefore, the link between Pol II transcription and RNA splicing ensures that the vast majority of pre-mRNAs enter the RNA processing pathway.

## Results

### Pre-mRNAs Synthesized by Pol II Are Spliced More Efficiently than pre-mRNAs Synthesized by T7 Polymerase

**Figure 1:**
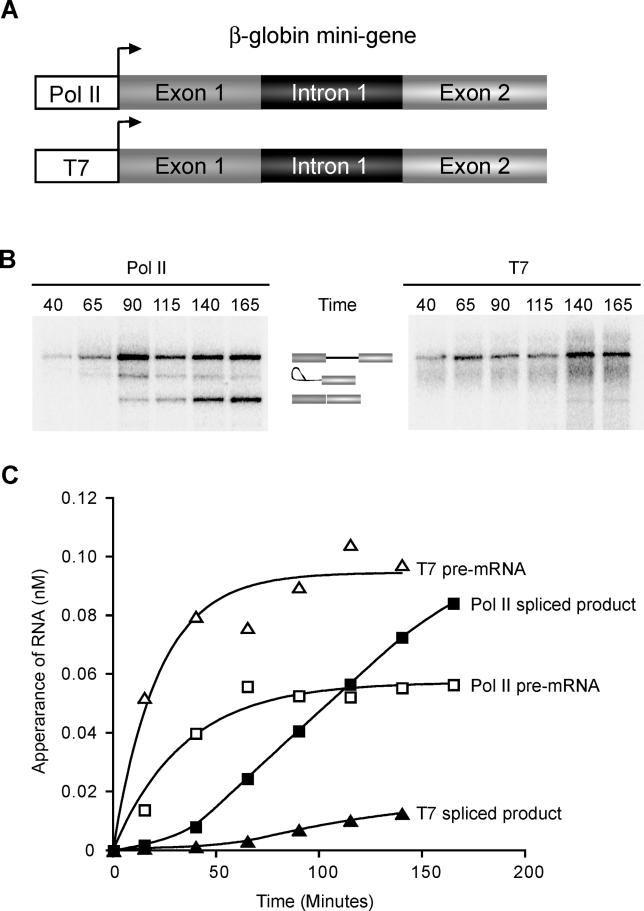
Linking Splicing to Pol II Transcription Increases the Efficiency of Pre-mRNA Splicing (A) Diagrams of the two templates used in the transcription/splicing assay, both contain the first exons of the *β-globin* gene. The template on the left is headed by the AdML Pol II promoter while the right template is headed by the T7 promoter. (B) Representative autoradiograms of the in vitro transcription/splicing assay appear below the diagrams. Pre-mRNA transcripts were either synthesized by Pol II (left) or T7 polymerase (right). Pre-mRNA transcript, spliced product, and intermediate lariat bands are indicated. (C) Quantitation of the data in (B). Molar concentration of RNA was determined by normalizing counts per min to a known concentration of radiolabeled RNA.

To examine the efficiency of pre-mRNA splicing when linked to Pol II transcription, we have established a highly efficient in vitro splicing assay that allows for RNA transcription and simultaneous splicing. HeLa cell nuclear extracts contain all components required for both processes; however, the most favorable conditions for either reaction are significantly different. By titrating nucleotide and magnesium concentrations, optimal conditions were derived to allow both processes to occur in the same reaction tube (unpublished data and Materials and Methods). In a first set of experiments, the efficiency of pre-mRNA splicing was compared between substrates generated by Pol II or by T7 polymerase. Pol II is a large polymerase (aggregate mass of > 500 kDa) with an in vivo elongation rate of ˜30 nucleotides per s [ 28]. It contains a CTD that is thought to recruit important components for RNA processing. T7 polymerase is a small (99 kDa) and slightly faster (elongation rate ˜40 nucleotides per s) polymerase devoid of a signature carboxy-terminus domain [ 29]. In vitro transcription/splicing reactions were initiated with two different polymerase-specific DNA templates encoding an identical *β-globin* minigene, the adenovirus major late (AdML) promoter for Pol II, and the T7 promoter for T7 RNA polymerase ( Figure 1A). Both templates generate pre-mRNAs as well as spliced products. However, a striking difference in splicing activity was observed ( Figure 1B). While approximately equal amounts of steady-state levels of pre-mRNA were produced, the conversion to intermediate lariat and spliced product was significantly more efficient for pre-mRNAs generated by Pol II ( Figure 1B and 1C). We conclude that Pol II-directed pre-mRNA synthesis increases spliceosomal efficiency.

### The In Vitro Rates of Splicing Are Equivalent for Pol II-Generated and Pre-Synthesized RNAs

**Figure 2:**
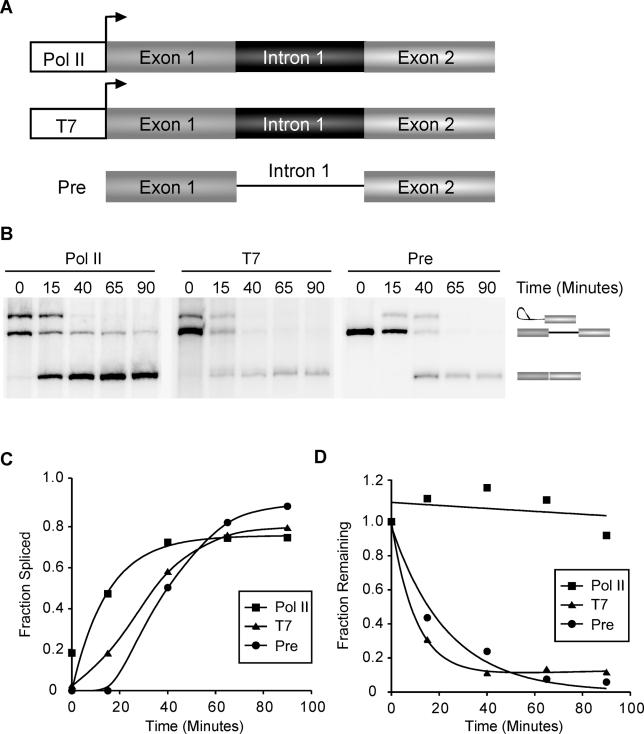
The Link between Pol II Transcription and Splicing Increases the Stability of Nascent Transcripts but Not the Rate of Splicing (A) Diagrams of the wt *β-globin* minigene DNA template with the Pol II promoter (top), the T7 promoter (middle), or the pre-synthesized RNA transcript (lower). (B) Representative autoradiograms show the time course of the transcription/splicing UTP chase experiment for Pol II- (left) or T7- (middle) generated transcripts, and the splicing profile of pre-synthesized pre-mRNAs (right). For the transcription/splicing UTP chase experiment, the zero time point refers to the initiation of the chase reaction through the addition of excess unlabeled UTP. Pre-mRNA transcript, spliced product, and intermediate lariat bands are indicated. The pre-synthesized pre-mRNA (right) is capped. (C) Quantitation of the data in (B) by computing the fraction spliced ([lariat] + [product])/ ([lariat] + [product] + [pre-mRNA]). (D) Quantitation of transcript degradation ([lariat] + [product] + [pre-mRNA]).

At the conditions used, transcription by either T7 polymerase or RNA Pol II generates similar amounts of RNA; however, the conversion of pre-mRNA to spliced product is efficient only when linked to transcription by Pol II. These observations suggest that the integration of Pol II transcription with pre-mRNA splicing either increases the maximal rate of splicing, or that the close association of the machineries leads to more efficient binding. To differentiate between these possibilities, we incorporated a nucleotide triphosphate (NTP) chase protocol along with the in vitro transcription/splicing assay. After incubation at reaction conditions for 20 min, an excess of uridine triphosphate (UTP) was added to quench the synthesis of radiolabeled pre-mRNAs. The fate of the labeled RNA that was generated during the initial pre-chase conditions was then evaluated independent of new pre-mRNA synthesis. The addition of excess UTP therefore uncouples splicing from RNA synthesis. The reactions were initiated with polymerase-specific DNA templates of the *β-globin* minigene, or with a pre-synthesized transcript of the same minigene ( Figure 2A). As illustrated in Figure 2B and 2C, the kinetics of intron removal were observed to be similar, regardless of the origin of the pre-mRNA. We measured a k _spl_ of 0.03/min, a value that is ˜10-fold slower than the rate measured in living cells for the identical intron removal event [ 30]. As expected from previous analyses, processing of pre-synthesized substrates in nuclear extracts is preceded by a short lag [ 31]. However, an analysis of the splicing efficiencies beyond the initial lag demonstrates that both conditions display similar splicing kinetics. These results demonstrate that the link between transcription and splicing does not increase the in vitro rate of intron removal.

Further inspection of the splicing profiles shows that the majority of the Pol II transcripts can be accounted for by their conversion into intermediate lariat or spliced product ( Figure 2B and 2D). On the other hand, pre-mRNAs generated by T7 polymerase degrade 10-fold more rapidly, regardless whether the RNA was generated in a T7 transcription/splicing reaction or whether it was generated in a separate synthesis reaction ( Figure 2B and 2D). For the pre-synthesized RNA, most of this degradation occurs during the first few min of incubation ( Figure 2D), a time period that coincides with a lag in splicing activity ( Figure 2C). We conclude that the increase in the efficiency of spliced product formation ( Figure 1) is not due to an increase in the rate of splicing, but rather due to a greater stability of the Pol II-generated transcript.

### The Presence of Splice Sites and Functional Spliceosomes Increases the Stability of Pol II Transcripts

**Figure 3:**
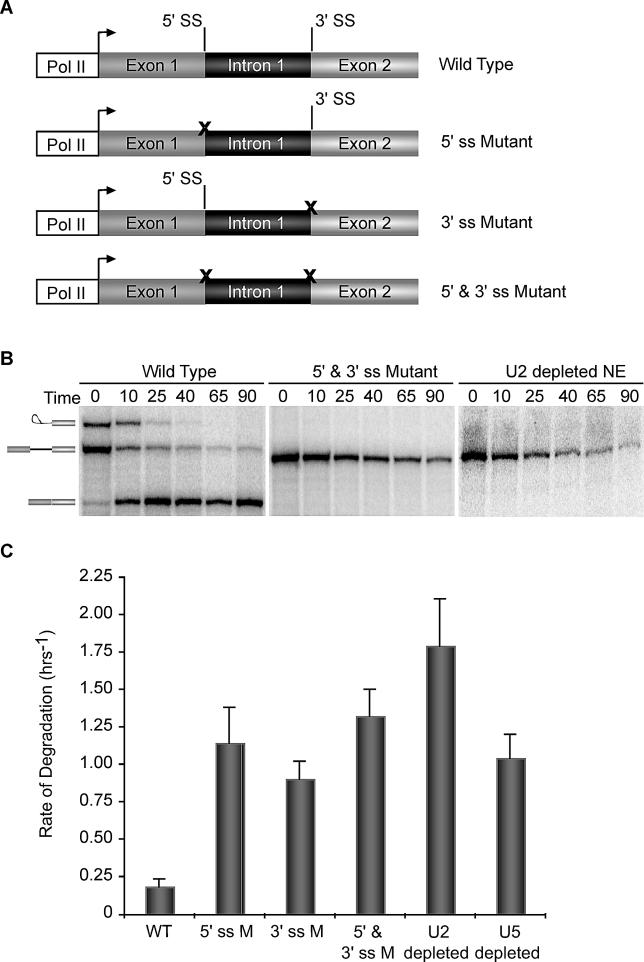
Functional Splice Sites Increase the Stability of Pol II Transcripts (A) Diagrams of DNA templates for wt (top) and mutant *β-globin* minigenes. The X indicates abolished splice sites. (B) Representative autoradiograms of the transcription/splicing chase experiments for the wt substrate (left), the double 5′ and 3′ splice site mutant (ss M) (middle), the mutant substrate (middle), and the wt substrate in extracts depleted of functional U2 snRNA (right). (C) Average rates of degradation observed for the Pol II transcripts wt, 5′ ss M, 3′ ss M, the double 5′ and 3′ ss M, wt substrate in U2 snRNA depleted extract, and wt substrate in U5 snRNA depleted extract.

Pol II transcripts are capped at the 5′ end by the sequential action of the capping enzyme Hce1 and the cap methyltransferase Hcm1. Thus, increased stability of Pol II-generated transcripts may be caused by protection from 5′ exonucleases. However, transcript stability may also be increased due to spliceosomal activity. To evaluate the contribution of splicing on transcript stability, we made use of the nucleotide chase protocol to measure the degradation profiles for various Pol II transcripts coding for the wild-type (wt) minigene, minigenes with an abolished 5′ or 3′ splice site ( Figure 3A), or an intronless minigene ( Figure S1). Compared to the wt minigene, a transcript containing abolished 5′ and 3′ splice sites displayed significantly higher rates of degradation ( Figure 3B). The quantitation of the degradation profiles shows that transcripts harboring two functional splice sites are 7.5-fold more stable than their mutant counterparts ( Figure 3C). Furthermore, the stability of an intronless RNA that was either generated by Pol II or pre-synthesized by T7 polymerase was observed to be similar at both reaction conditions ( Figure S1). Thus, transcription by Pol II alone is not sufficient to guarantee protection from degradation. Rather, functional splice sites are required for the observed stability.

An additional approach was taken to evaluate the influence of splicing on the stability of the newly synthesized pre-mRNA. Rather than mutating the splice sites of the test substrates, the activity of the splicing machinery was targeted. Antisense oligonucleotides were designed to interact with either U2 snRNA [ 32] or U5 snRNA [ 33]. Through the addition of RNase H, these antisense oligonucleotides mediated essentially complete inactivation of the spliceosome (unpublished data and Figure 3B). The stability of the wt minigene was then assayed using the transcription/splicing NTP chase protocol described above. As predicted from the mutational studies, depletion of functional spliceosomes resulted not only in a loss of splicing activity, but, more importantly, in a significant increase in the degradation rate of the newly synthesized pre-mRNA ( Figure 3B and 3C). Interestingly, targeting U2 or U5 snRNA destabilized the pre-mRNA to a similar degree. These results suggest that the assembly of the complete spliceosome is required to maintain pre-mRNA stability. We conclude that the assembly of the spliceosome onto functional splice sites increases the stability of pre-mRNAs generated by Pol II.

### The Link between Splicing and Pol II Transcription Ensures That Pre-mRNAs Enter the Processing Pathway rather than the Degradation Pathway

**Figure 4:**
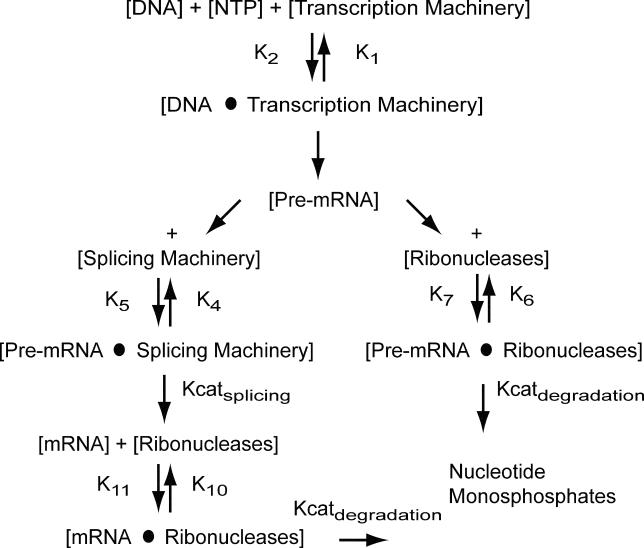
Minimal Kinetic Scheme of the In Vitro Reaction Linking Pol II Transcription with Pre-mRNA Splicing The diagram shows a series of dissociation and association constants for the formation of complexes between the DNA template and the transcription machinery, between the pre-mRNA and ribonucleases or the splicing machinery, and between the spliced mRNA and ribonucleases. The catalytic rate constants describe the conversion of NTPs to pre-mRNA for transcription, the conversion of pre-mRNA to mRNA for splicing, and the conversion of pre-mRNA or mRNA to nucleotide monophosphate for ribonuclease digestion.

**Figure 5:**
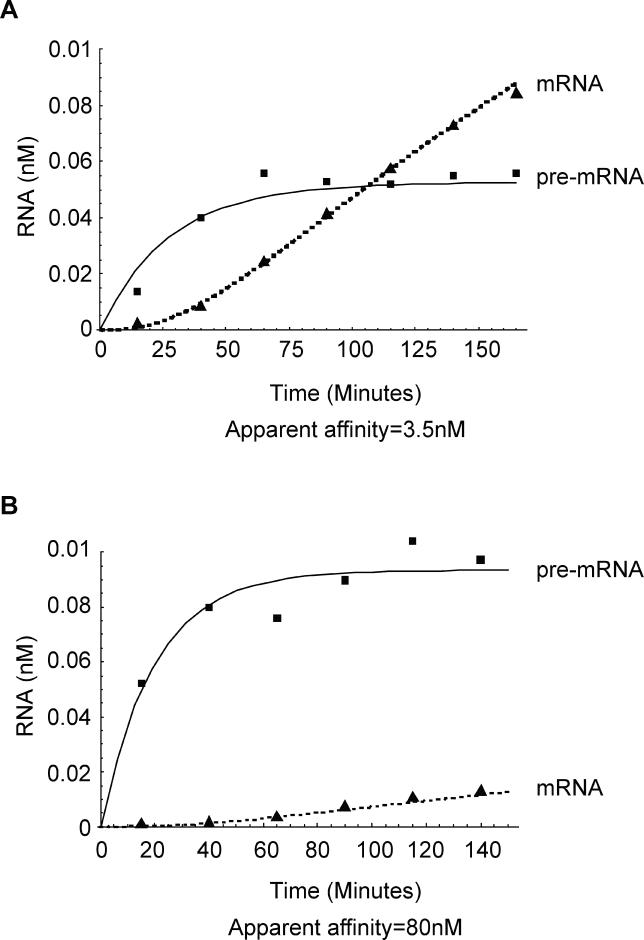
The Link between Pol II Transcription and Pre-mRNA Splicing Increases the Affinity of Splicing Factors to the Nascent Pre-mRNA A mathematical model is used to describe the basic reactions involved in the coupled transcription/splicing reaction. The splicing profiles obtained from RNAs generated by Pol II (A) or by T7 polymerase (B) were fit to the mathematical prediction by testing various affinities (K _m_) between the splicing machinery and the pre-mRNA. (A and B) represent the best fit for either reaction. Squares represent pre-mRNA and triangles represent spliced mRNA. The solid line represents the mathematical prediction for the accumulation of pre-mRNA based on the K _m_ value indicated below each graph. The dotted line represents the mathematical prediction for the accumulation of spliced mRNA based on the K _m_ value indicated below each graph.

The above experiments suggest that Pol II-directed pre-mRNA synthesis accelerates spliceosomal assembly and activity by increasing the local concentration of splicing factors in the vicinity of the newly synthesized pre-mRNA. As a consequence, more pre-mRNAs enter the processing pathway compared to assays that measure the processing of a pre-synthesized transcript alone. To further evaluate this hypothesis, we developed a computational approach to model the continuous RNA synthesis and processing reactions as they occur in the in vitro transcription/splicing assay. We derived a minimal kinetic scheme that describes the fate of each pre-mRNA, from synthesis to processing, and, finally, to degradation ( Figure 4). A mathematical model for the transcription/splicing system was built by defining each step of the reaction (transcription, splicing, and degradation) with a set of differential equations representing association, dissociation, and catalytic rate constants. The suite of individual reactions was then translated by the program kMech/Cellerator [ 34] to determine the flux of reaction intermediates and concentrations of products. With the exception of the observed binding constant of the spliceosome to the pre-mRNA, all kinetic parameters described in the reaction scheme ( Figure 4) were either determined experimentally, or estimated from literature values ( Table S1). For example, Figure 2 demonstrates that the observed k _spl_ of splicing is 0.03/min, independent of a link to transcription. In addition, the active concentration of spliceosomes at the conditions used was estimated by independent titration experiments to be ∼5 nM ( Figure S2). As a result, we were able to fit the pre-mRNA/mRNA profiles shown in Figure 1B to the mathematical model by altering only a single variable, the apparent affinity of the spliceosome for the pre-mRNA. The best fit of the Pol II-dependent splicing profile with the mathematical predictions was observed at an apparent K _m_ of 3.5 nM ( Figure 5A). By contrast, when linked to T7 transcription, the apparent affinity of the spliceosome to the newly synthesized pre-mRNA is about 20-fold lower ( Figure 5B). Thus, pre-mRNAs generated by Pol II are much more likely to associate with the spliceosome compared to pre-mRNAs generated by T7 polymerase. We conclude that the association of Pol II transcription and pre-mRNA processing ensures efficient transfer from the transcription machinery to the RNA processing machinery, rendering pre-mRNAs less accessible to nucleases.

### The Relative Synthesis of Competing Splice Sites Influences Alternative 3′ Splice-Site Choice

**Figure 6:**
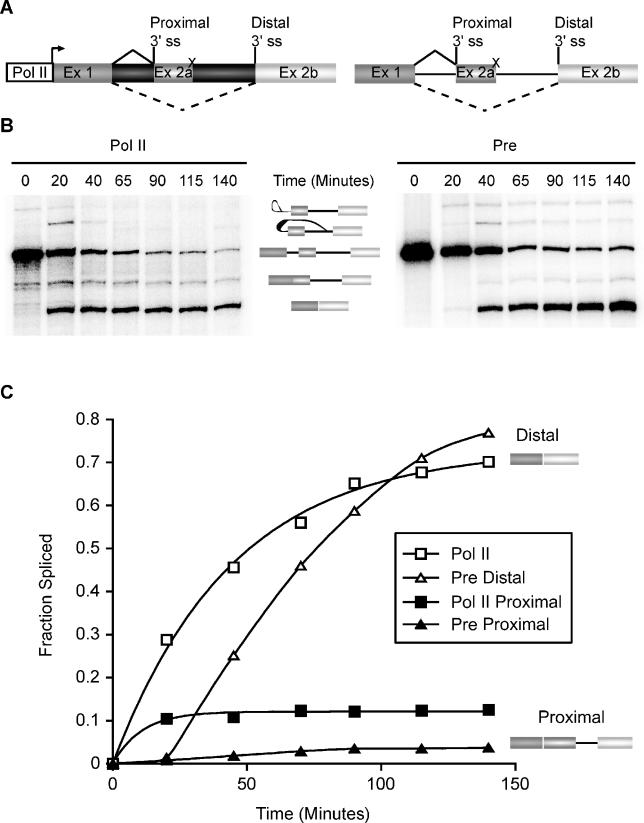
The Link between Pol II Transcription and Splicing Influences Alternative 3′ Splice-Site Choice (A) Diagrams of the *wt14* minigenes with duplicated 3′ splice sites, either as a DNA template preceded by a Pol II promoter (left) or the pre-synthesized RNA transcript (right). The proximal exon (exon 2A) does not contain a 5′ splice site, is significantly shorter than the distal exon, and lacks exonic splicing enhancers. (B) Representative autoradiograms show the time course of the transcription/splicing UTP chase experiment (left) and the splicing profile of pre-synthesized RNA transcripts (right). The pre-mRNA transcript, spliced products, and intermediate lariat bands are indicated. (C) Quantitation of distal and proximal spliced product formation from (B).

It has been proposed that the kinetics of transcription can influence alternative splice-site choice [ 7]. To test whether the association of Pol II transcription and pre-mRNA splicing affects alternative splicing patterns, we evaluated splice-site selection of a 3′ splice-site duplication substrate. This pre-mRNA is identical to the wt14 substrate recently characterized that contains a common upstream exon and two competing downstream exons of different length [ 35] ( Figure 6A). The splicing profile of wt14 was determined using either our transcription/splicing assay or by the addition of pre-synthesized pre-mRNAs to nuclear extracts. In agreement with previous observations for the substrate wt14, distal splicing is much more preferred over proximal splicing ( Figure 6B). This is because the proximal exon lacks functional exonic splicing enhancers [ 35]. However, a significant difference in the extent of proximal splice-site activation was observed between the reaction conditions used. When splicing was linked to transcription by Pol II, the proximal to distal ratio was observed to be 1/6 ( Figure 6B and 6C). By contrast, splicing of a pre-synthesized substrate resulted in a proximal to distal ratio of 1/25 ( Figure 6B and 6C). Thus, linking transcription to splicing increases proximal splice-site usage by 5-fold. This change in alternative splice-site choice is polymerase-specific because no detectable proximal splicing was observed for pre-mRNA substrates that were linked to transcription by T7 polymerase (unpublished data). Interestingly, when linked to transcription by Pol II the majority of proximal splicing occurs within the first few min of the experiment (< 20 min) and subsequent splicing is mainly directed to the distal site ( Figure 6B, left lane). It is possible that this initial burst in proximal splicing derives from partially synthesized pre-mRNA transcripts for which the distal site has not yet been transcribed. Once transcription is complete and higher levels of full-length transcripts are available, the ratio of splicing to the competing sites might be similar to that observed for the pre-synthesized RNA. We conclude that the process of Pol II transcription influences alternative splice-site choice.

The observations described above support the notion that the relative synthesis of competing exons influences alternative splice-site pairing. Indeed, in agreement with previous work [ 21, 24], it was shown that delaying the synthesis of a competing downstream exon by lengthening the intron resulted in increased levels of upstream splice-site selection ( Figure S3).

## Discussion

Recent analyses have demonstrated that spliceosomal assembly can occur co-transcriptionally, before the RNA is released from the Pol II [ 36, 37]. Although biochemical evidence strongly supports direct interactions between Pol II and RNA processing factors involved in 5′ capping and polyadenylation, little is known how the physical and temporal association of Pol II transcription and the splicing machinery ensures efficient pre-mRNA splicing. To permit biochemical analysis, we have established an in vitro transcription/splicing assay that recapitulates transcription and intron removal in a test tube. This assay was used to perform a series of kinetic analyses to demonstrate that the association of pre-mRNA splicing with transcription by Pol II significantly increases the half-life of the RNA transcript, and that the process of transcription influences alternative splice-site choice.

### The Link between Transcription and Splicing Ensures Efficient Transfer of the Newly Synthesized Pre-mRNA to the Splicing Machinery

A comparison of the splicing profiles from pre-synthesized or Pol II-transcribed test RNAs demonstrated a significant increase in stability of intron-containing RNAs generated by Pol II. This observation suggests that a major advantage of integrating splicing and transcription is avoidance of nucleases, thus ensuring that the majority of pre-mRNAs enter the RNA processing pathway. To protect the RNA from exonucleases, the 5′ and 3′ ends of pre-mRNAs are modified. As has been demonstrated, these events are orchestrated through direct interactions of the appropriate RNA processing factors with the phosphorylated form of the CTD (reviewed in [ 9– 12]). To deny access of endonucleases, the newly synthesized pre-mRNA must associate with competing RNA binding proteins, such as spliceosomal proteins or hnRNPs. Our analysis of the Pol II transcription/splicing reaction indicates that intron-containing RNAs generated by Pol II are much more likely to be accessed by spliceosomal factors than intron-containing RNAs generated by T7 polymerases ( Figure 3). In analogy to capping and polyadenylation, it is possible that the CTD of Pol II increases the local concentration of RNA splicing factors in the vicinity of the exiting RNA. Significantly, the increase in RNA stability is dependent on the presence of functional splice sites and a functional spliceosome ( Figure 3). Thus, fewer pre-mRNAs are degraded before the completion of spliceosomal assembly, and the majority of synthesized RNAs are carried over to the next step of the gene expression pathway. It is possible that the observed protection results from the initial recognition of splicing signals during exon definition, when U1 snRNP and U2AF associate with the pre-mRNA. Alternatively, stabilization of the transcript might require the assembly of the mature spliceosome. The fact that the stability of the test substrate was equally compromised when U2 snRNA or U5 snRNA were targeted by antisense oligonucleotides and RNase H ( Figure 3) argues strongly against the possibility that early assembly is sufficient for the observed pre-mRNA protection. Rather, protection depends on the incorporation of functional tri-snRNP, suggesting that later spliceosomal assembly steps ensure increased stability. Taken together, the experimental results combined with the mathematical modeling of continuous RNA synthesis and processing support the concept that the interaction and synchronous timing between Pol II transcription and pre-mRNA splicing significantly protect the newly synthesized RNA from degradation.

In higher eukaryotes, splice sites and regulatory sequences, such as exonic splicing enhancers and silencers, are not highly conserved [ 38, 39]. These observations suggest that interactions between the pre-mRNA and spliceosomal components are more dependent on high concentrations of binding partners than on high affinity. It is therefore likely that the proposed increase in the local concentration of RNA splicing factors in the vicinity of newly synthesized RNAs contributes to relaxing the evolutionary strain to maintain high levels of the conservation among splice sites and other RNA elements.

When Pol II links pre-mRNA splicing to transcription, we observed robust intron removal. However, when the identical RNA was generated by T7 polymerase, splicing efficiency markedly dropped ( Figure 1). These results agree quantitatively with recent reports comparing the splicing of Pol II- and T7 polymerase-generated pre-mRNAs [ 35, 40], although different salt and metal ion conditions were used [ 40].

### The Link between Transcription and Splicing Influences Alternative Splice-Site Choice

The results obtained from the analysis of alternative 3′ splice-site choice demonstrates that pre-mRNAs generated by Pol II are more likely to select the proximal splice site compared to pre-synthesized RNA substrates ( Figure 6). These observations are consistent with the hypothesis that the kinetics of transcription influence splice-site choice [ 7, 27]. The model suggests that the most proximal 5′ splice site benefits from the fact that it is synthesized prior to all other competing splice sites. This difference in the timing of synthesis could contribute to the preferential pairing of proximal splice sites [ 41]. However, it is also possible that the increased selection of the proximal splice site originates from differences in 5′ end modifications, or in the formation or prevention of RNA secondary structures that interfere or promote the recognition of the proximal splice site. Further support for the concept of a temporal advantage to a proximal splice site was provided by experiments designed to delay the relative synthesis of competing exons by expanding the intron separating them [ 24] (Figure S4). A linear increase in the fraction of internal exon inclusion was observed upon increasing the intron length separating the competing exons. In agreement with such an interpretation are the results obtained from an EST database analysis that demonstrated that the length of the flanking intron influenced the probability of an exon to undergo alternative splicing [ 42]. Longer introns correlated with a higher probability of alternative splicing. These results demonstrate that the relative synthesis of competing exons can influence splice-site choice.

### How Are Transcription and Pre-mRNA Splicing Linked?

Photo-crosslinking studies and crystallography have shown that the nascent RNA exits the polymerase in the vicinity of the CTD [ 43, 44]. In addition, some RNA processing factors associated with pre-mRNA splicing, including splicing factor Prp40 [ 45], interact with the phosphorylated form of the CTD, and Pol II CTD phosphorylation is required for efficient pre-mRNA splicing and 3′ end formation [ 46]. Moreover, the CTD of Pol II was shown to stimulate splicing independent of its effect on capping or polyadenylation [ 47]. These observations support a model for the coupling of Pol II transcription and pre-mRNA splicing. As transcription elongation occurs, splicing factors transfer from the phosphorylated CTD of Pol II onto the nascent transcript. However, due to the lack of direct physical evidence, it is currently unclear whether splicing and transcription are functionally coupled. That is, does pre-mRNA splicing require an activity that is provided only through Pol II transcription? Beyond the obvious requirement of generating the pre-mRNA substrate, no such activity other than the CTD is yet known.

Recent cross-linking/immunoprecipitation experiments have described the co-transcriptional assembly of splicing components onto nascent pre-mRNAs, demonstrating that the assembly of the spliceosome, and possibly intron excision, occurs before Pol II terminates transcription [ 36, 37]. Considering the splicing kinetics observed in our transcription/splicing assay, it appears unlikely that intron removal is completed before the transcription machinery reaches the end of the DNA template. However, pre-mRNA splicing is not always carried out co-transcriptionally. As was shown for the Balbiani ring genes of *Chironomus tentans,* upstream introns are excised while transcription is in progress; yet, downstream introns were removed post-transcriptionally [ 48]. Given the considerable variation of splice-site strength and intron length, both of which influence the kinetics of spliceosomal assembly and the time span between the synthesis of competing exons, it is expected that a significant fraction of exons will not be processed co-transcriptionally.

The results presented here underscore the importance of integrating gene expression processes, spatially and temporally. They support the growing concept that nuclear processing events are not independent and compartmentalized, but rather linked in a continuous trajectory. In this scenario, processing factors recruited at the DNA level are handed off to the newly synthesized RNA. It will be of particular interest to determine whether the link of transcription and pre-mRNA splicing merely increases the concentration of splicing factors or whether a higher order gene expression complex selectively deposits splicing factors onto the pre-mRNA. No doubt, the in vitro transcription/splicing assay described here will be a valuable tool in addressing these important questions.

## Materials and Methods

### DNA templates.

To generate the constructs used in the transcription/splicing assays, a fragment containing exons one through two of *β-globin* was excised at the 5′ end with *PvuII* and at the 3′ end with *EcoRV* and inserted into the SP73 plasmid, downstream of the T7 transcription start site. The same *β-globin* minigene fragment was inserted into the *PflmI* site (downstream of the RNA Pol II transcription start site) of the pMLP-U-A plasmid containing the AdML promoter (courtesy of Brenton Graveley). DNA templates containing the promoter and encoding regions of the RNAs were generated by PCR from these constructs and subsequently digested with *EcoRI* or *MluI.* To make the constructs used to evaluate the 5′ splice site, a PCR-generated product beginning immediately downstream of the 5′ splice site of exon one continuing through exon two of *β-globin,* was inserted downstream of a test 5′ splice site containing one of two 5′ splice sites, strong (5′-CAG| GTAAGTCA-3′) or abolished (5′-GTA| CTTCTACA-3′). This fragment was blunt-ended and inserted into the *PflmI* site of the same pMLP-U-A AdML promoter plasmid. Constructs used to evaluate the 3′ splice site, the SP73 *β-globin* minigene constructs containing tandem repeats of the 3′ splice site, were described previously [ 41]. These minigene constructs were also excised using *PvuII* and *EcoRV* and inserted into *PflmI* site of the pMLP-U-A plasmid. DNA templates containing the promoter and encoding regions of the RNAs were generated by PCR from these constructs and subsequently digested with either *EcoRI* or *HindIII.*

### RNAs.

DNA templates containing the T7 promoter and encoding regions of the *β-globin* minigenes were generated by PCR using a T7 forward primer, which anneals at the transcription initiation start site (of RNA Pol II) in the pMLP-U-A *β-globin* minigene constructs and a SP6 reverse primer, which anneals downstream of the minigenes. PCR products were subsequently digested with *EcoRI, MluI,* or *HindIII.* Pre-synthesized ^32^P-labeled RNA transcripts were synthesized with T7 RNA polymerase in the presence of 2 mM m ^7^G (5′) ppp (5′) G cap analog and resolved and purified on 6% denaturing PAGE [ 31].

### In vitro transcription/splicing assay.

Reactions were carried out in 50% nuclear extract [ 49] containing PCR-generated DNA template, 0.2 mM CTP and GTP, 25 μM UTP, 0.2 μM ^32^P-UTP, 1.0 mM ATP, 20 mM creatine phosphate, 4 mM MgCl _2_, and incubated at 30 °C for a determined time course indicated in figures. To approximate the same RNA yield, T7 RNA polymerase was added to reactions containing DNA templates at a final concentration of 0.07 units/μL (˜3 nM). The amount of T7 polymerase added to the reactions was determined by titration experiments (unpublished data). Following incubation, reactions were digested with proteinase K, phenol chloroform extracted, ethanol precipitated, resolved on 8% denaturing PAGE, and analyzed by PhosphorImager analysis as previously described [ 50].

### In vitro transcription/splicing “cold” UTP chase.

Reactions were carried out as described for the transcription/splicing assay, except that after a 20-min incubation, 3 mM UTP was added to chase the ^32^P-labeled RNA Pol II transcript. Time points were taken immediately after the addition of the chase (defined as the zero chase time point) and as indicated in Figures 2, 3, and 6.

### In vitro splicing assay.

Conditions were the same as described for the in vitro transcription/splicing assay except that pre-synthesized ^32^P-labeled RNAs were used as a splicing template and ^32^P-labeled UTP was not added.

### Ribonuclease H digestion of U2 or U5 snRNA.

Selective targeting of U2 [ 32] or U5 snRNA [ 33] followed protocols previously described. RNase H-mediated depletion of functional snRNPs was carried out in 1.5 mM ATP, 5 mM creatine phosphate, 1.6 mM MgCl _2_, 40 μM anti-snRNA DNA oligonucleotides, and 93% nuclear extract at 30 °C for 1 h. This treatment resulted in splicing deficient nuclear extract (unpublished data). The depleted extracts were then used to carry out splicing reactions at identical experimental conditions as described above. The sequences of DNA oligonucleotide used were 5′- GGCCGAGAAGCGAT-3′ (against U2 snRNA) and 5′- TTAAGACTCAGAGTTGTTCC- 3′ (against U5 snRNA).

### Cell culture and transfections.

HeLa cells were grown in MEM supplemented with 10% (vol/vol) FBS and 2 mM glutamine and plated 1 × 10 ^5^ to 3 × 10 ^5^ cells/well 20–24 h before transfection in 24-well plates. 0.5 μg of the *SMN2* minigenes were transfected using LipoFectamine 2000 and incubated for 24 h. Total RNA was extracted and then isolated as previously described [ 51]. Reaction products were fractionated on a 2% agarose gel. The resulting bands were visualized and quantitated using a gel-doc imager (BioRad, Hercules, California, United States). The exon 7 inclusion efficiency was calculated by computing the pixel count of exon 7 included over the sum of the pixel counts of exon 7 included and excluded. Transfection experiments were repeated at least five times.

### Mathematical modeling of transcription and pre-mRNA splicing.

The mathematical model of transcription/splicing was built using kMech/Cellerator [ 34]. kMech is a Cellerator [ 52] language extension that describes a suite of enzyme reaction mechanisms. Each enzyme mechanism is parsed by kMech into a set of fundamental association-dissociation reactions that are translated by Cellerator into ordinary differential equations (ODEs) that are numerically solved by Mathematica ^TM^ [ 34]. The minimal reaction scheme for the transcription/pre-mRNA splicing reaction is shown in Figure 4. The model consists of three major reactions: Transcription of DNA, splicing of pre-mRNA, and degradation of pre-mRNA and mRNA. This reaction model can be represented by the following six kMech/Cellerator reactions: The first reaction represents “transcription of DNA” modeled by the kMech generalized BiBi (two-substrate, two-product) reaction. NTP represents the four nucleotides required for transcription. The second reaction represents spliceosome binding to the pre-mRNA modeled by the Cellerator simple catalytic model. The third reaction represents mRNA generation and release from the spliceosome/pre-mRNA complex modeled by the Cellerator simple catalytic model. The fourth to sixth reactions represent pre-mRNA and mRNA degradation by RNase modeled by the Cellerator simple catalytic model. The above reaction steps were translated by Cellerator into 13 ODEs with 15 rate constants that describe the rates of change of 13 reactants involved in the model. The forward rate constants and reverse rate constants, usually not available experimentally, are approximated by Lambda approximation method from kinetic measurements *(K _m,_ k _cat_)* of enzymes [ 34].

The model described above, kMech and Cellerator, are available at the Institute for Genomics and Bioinformatics, University of California, Irvine, Irvine, California, United States ( http://www.igb.uci.edu/servers/sb.html). They are free of charge to academic, United States government, and other nonprofit organizations.

## Supporting Information

Figure S1 Pol II Transcription Does Not Increase the Stability of a Nascent Pol II Transcript Void of Splicing Signals (A) Diagrams of the intronless DNA template and the pre-synthesized intronless RNA transcript. (B) Autoradiogram of the transcription/splicing UTP chase experiment of intronless RNA generated from the Pol II template (left lane) compared to the degradation profile of a pre-synthesized transcript (right lane). The UTP chase was initiated after 20 min by adding saturating amounts of unlabeled UTP. The migration of the intronless RNA transcript is indicated on the right. (C) Quantitation of the data in (B) showing the degradation profile of the intronless transcripts over time.
(495 KB PDF)

Figure S2 The Kinetics of Transcription Influence Exon Inclusion (A) Diagram of the *SMN2* minigene, indicating the site of nucleotide insertion. (B) Representative autoradiogram displaying the exon 7 inclusion ratio for *SMN2* constructs containing none or multiple 500 nucleotide intron 7 inserts. Band representing exon 7 inclusion or exclusion are indicated on the right. The fraction exon 7 inclusion is shown below each lane. (C) Quantitation of the data shown in (B) as a function of insert length.
(753 KB PDF)

Figure S3 Titration of Spliceosomes To estimate the concentration of active spliceosomes, increasing amounts of pre-mRNA (0.2–200 nM) were incubated at standard reaction conditions. The splicing rates determined from each of the time course reactions are plotted against the concentration of pre-mRNA added. Saturation of active spliceosome is indicated by the drop in the observed rate of intron removal, corresponding to ∼5 nM.
(169 KB PDF)

Table S1 Summary of the Rate and Dissociation Constants Used in the Mathematical Model (59 KB DOC)

## Abbreviations

AdML: adenovirus major late
CTD: C-terminal domain
NTP: nucleotide triphosphate
Pol II: RNA polymerase II
UTP: uridine triphosphate
wt: wild-type
