# Supplementary material for: Linking Splicing to Pol II Transcription Stabilizes Pre-mRNAs and Influences Splicing Patterns
Source: PLoS Biol. 2006 May 2;4(6):e147. doi: 10.1371/journal.pbio.0040147 (PMC1450099; doi:10.1371/journal.pbio.0040147)
Supplement: Supplementary file 1 [file pbio.0040147.sg001.pdf]

A

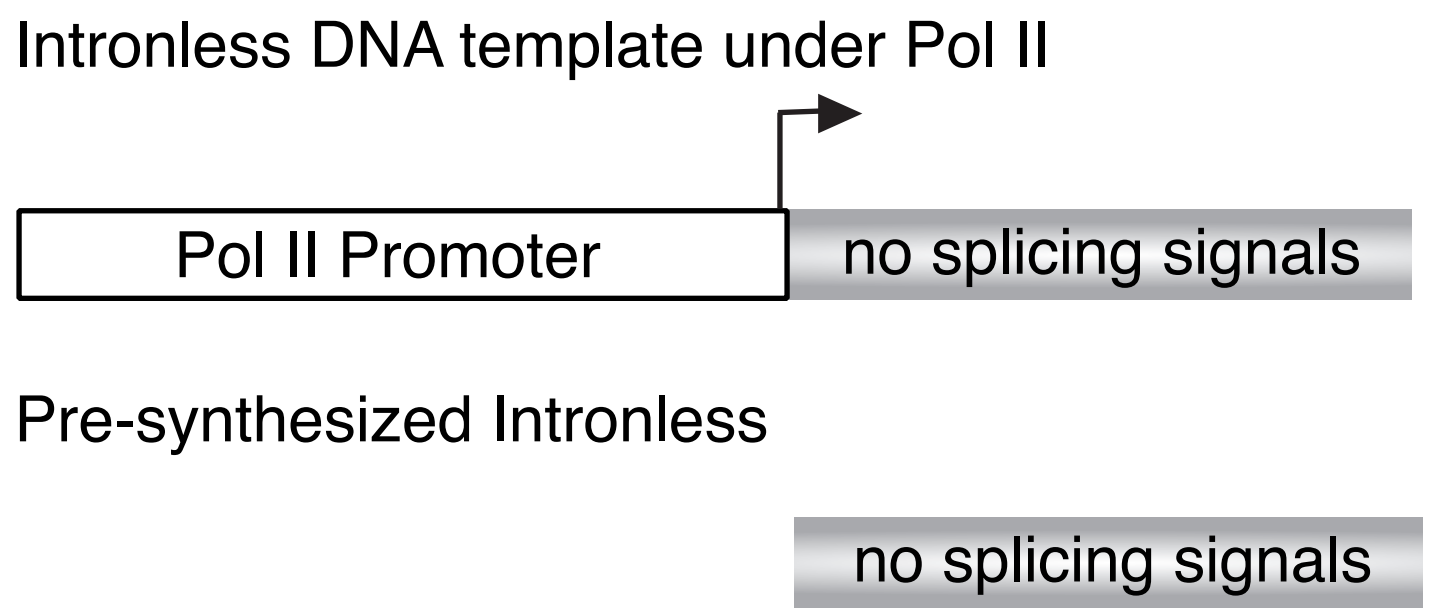
Intronless DNA template under Pol II
Pol II Promoter
no splicing signals
Pre-synthesized Intronless
no splicing signals

B

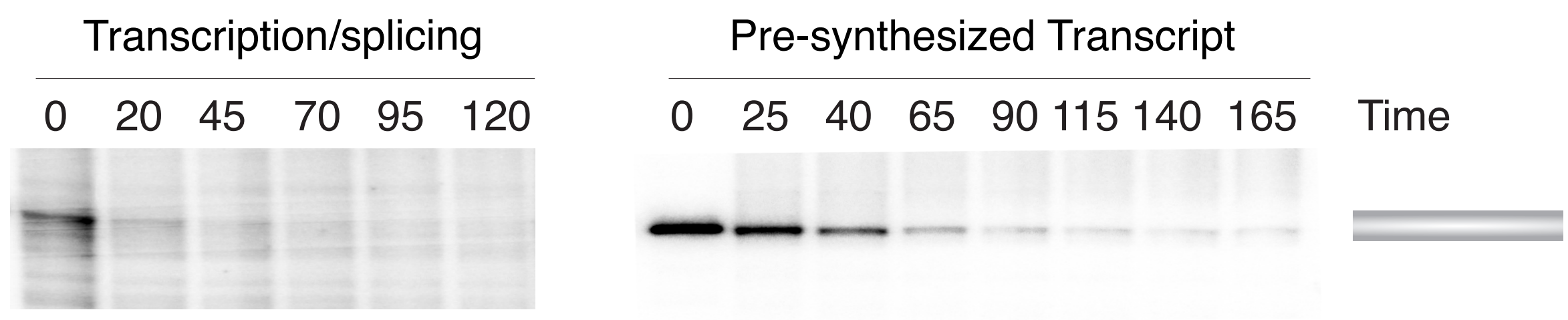
Transcription/splicing
0 20 45 70 95 120
Pre-synthesized Transcript
0 25 40 65 90 115 140 165
Time

C

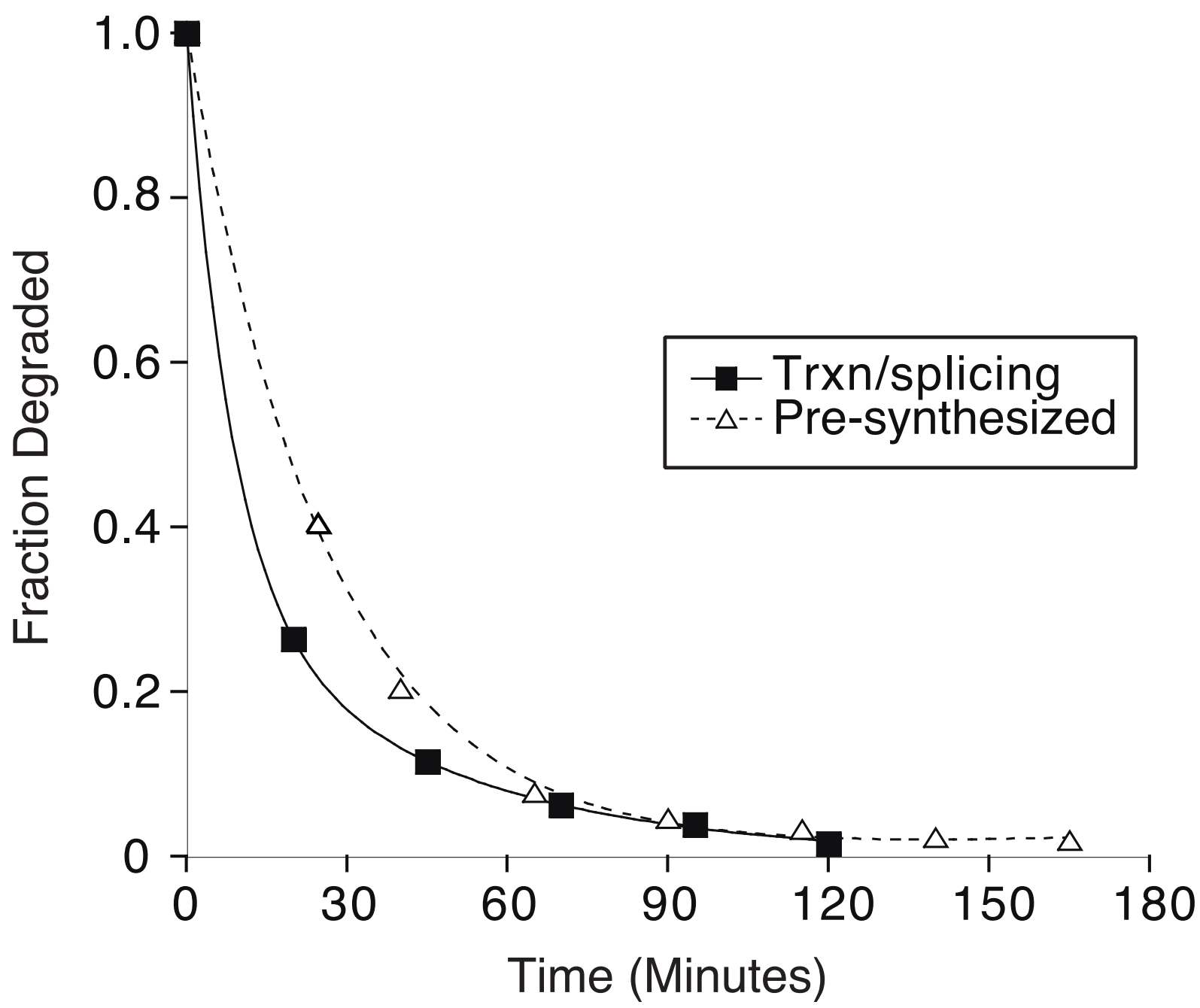
Fraction Degraded
1.0
0.8
0.6
0.4
0.2
0
0 30 60 90 120 150 180
Time (Minutes)
Trxn/splicing
Pre-synthesized
