# Supplementary material for: Linking Splicing to Pol II Transcription Stabilizes Pre-mRNAs and Influences Splicing Patterns
Source: PLoS Biol. 2006 May 2;4(6):e147. doi: 10.1371/journal.pbio.0040147 (PMC1450099; doi:10.1371/journal.pbio.0040147)
Supplement: Supplementary file 2 [file pbio.0040147.sg002.pdf]

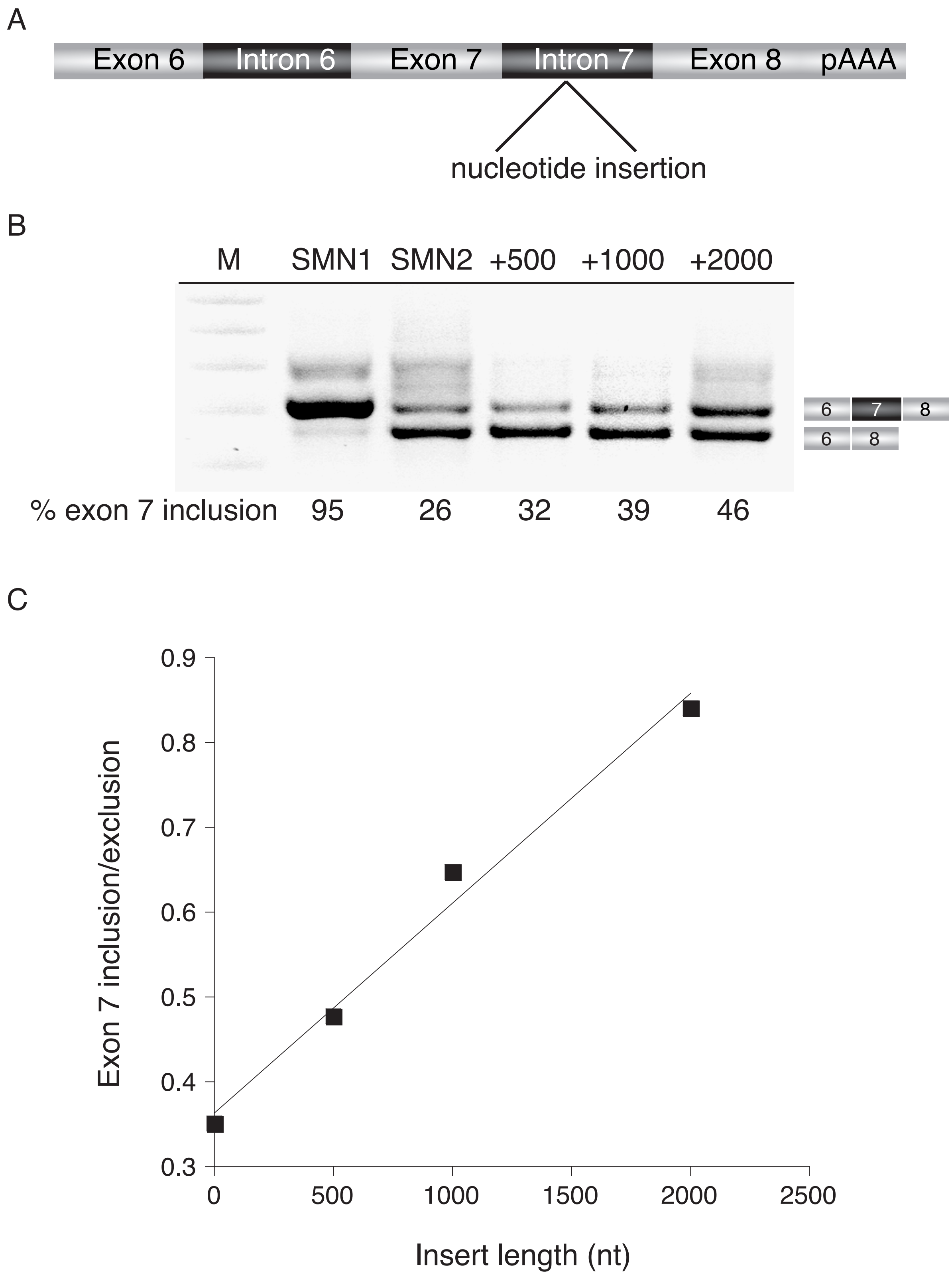
A
Exon 6
Intron 6
Exon 7
Intron 7
Exon 8
pAAA
nucleotide insertion
B
M
SMN1
SMN2
+500
+1000
+2000
6 7 8
6 8
% exon 7 inclusion
95
26
32
39
46
C
Exon 7 inclusion/exclusion
0.9
0.8
0.7
0.6
0.5
0.4
0.3
0
500
1000
1500
2000
2500
Insert length (nt)
