# Supplementary material for: Linking Splicing to Pol II Transcription Stabilizes Pre-mRNAs and Influences Splicing Patterns
Source: PLoS Biol. 2006 May 2;4(6):e147. doi: 10.1371/journal.pbio.0040147 (PMC1450099; doi:10.1371/journal.pbio.0040147)

Supporting Figure 3

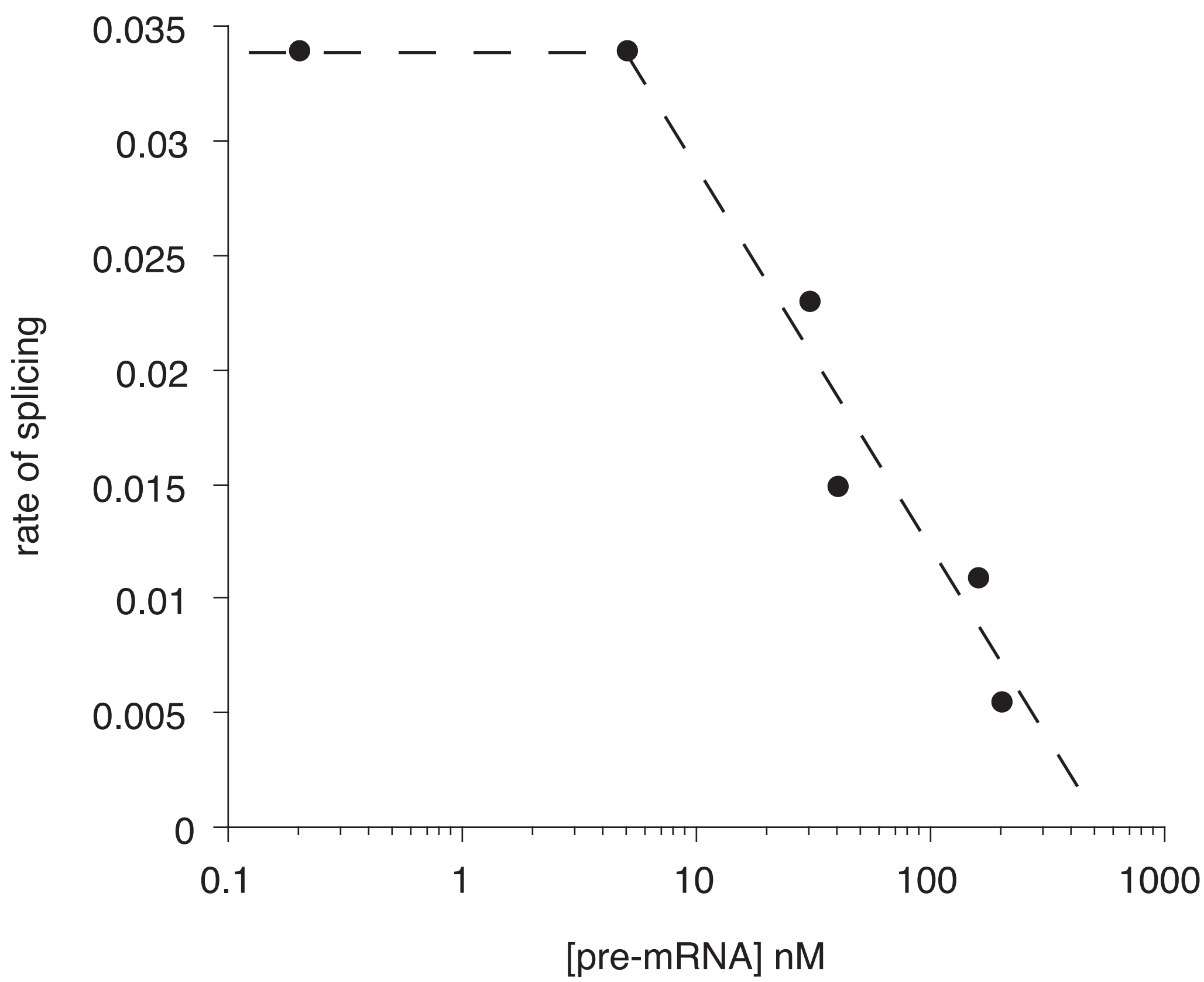

Supplement: Supplementary file 3 [file pbio.0040147.sg003.pdf]
