# Supplementary material for: Linking Splicing to Pol II Transcription Stabilizes Pre-mRNAs and Influences Splicing Patterns
Source: PLoS Biol. 2006 May 2;4(6):e147. doi: 10.1371/journal.pbio.0040147 (PMC1450099; doi:10.1371/journal.pbio.0040147)
Supplement: Supplementary file 4 [file pbio.0040147.st001.doc]

**Supporting Table 1.**

Summary of the rate and dissociation constants used in the mathematical model.

|  | Pol II | T7 |
| --- | --- | --- |
| K_m_ DNA•Txn | 40 nM (Fig.1 based on flux) | 1400 nM [1] |
| K_m_ NTP•Txn | 1000 nM [2] | 600000 nM [3] |
| k_cat_ Txn | 0.1 min^-1^ (promoter clearance is rate limiting) [4] | 4.5 min^-1^ (reflects 550 nt x 2580 nt/min^-1^) [5] |
| K_m_ preRNA•Spl | 3.5 nM (this study) | 80 nM (this study) |
| k_cat_ Spl | 0.03 min^-1^ (Fig. 2) | 0.03 min^-1^ (Fig. 2) |
| K_m_ preRNA•RNase | 2000000 nM (based on RNase A) [6] | 2000000 nM (based on RNase A) [6] |
| k_cat_ RNase | 30000 min^-1^ (based on RNase A) [6] | 30000 min^-1^ (based on RNase A) [6] |
| K_m_ mRNA•RNase | 30000000 nM (Fig. 2 and based on RNase A) [6] | 30000000 nM (Fig. 2 and based on RNase A) [6] |
| k_cat_ RNase | 30000 min^-1^ (based on RNase A) [6] | 30000 min^-1^ (based on RNase A) [6] |
| [DNA] | 12 nM (experimental condition) | 12 nM (experimental condition) |
| limiting [NTP] | 25000 nM (experimental condition) | 25000 nM (experimental condition) |
| [Txn]^1^ | 0.025 nM (Fig. 1 based on flux) | 3 nM (experimental condition) |
| [Spl]^2^ | 5 nM (Supplementary Fig. 3) | 5 nM (Supplementary Fig. 3) |
| [RNase] | 3.5 nM (Fig. 2 and based on RNase A) [6] | 3.5 nM (Fig. 2 and based on RNase A) [6] |

^1^Concentration of transcription machinery

^2^Concentration of splicing machinery
